# Functions of the Three Common Fungal Extracellular Membrane (CFEM) Domain-Containing Genes of *Arthrobotrys flagrans* in the Process of Nematode Trapping

**DOI:** 10.3390/microorganisms13092001

**Published:** 2025-08-27

**Authors:** Tingting Shi, Xiaotong Deng, Yu Zhang, Guohong Li

**Affiliations:** State Key Laboratory for Conservation and Utilization of Bio-Resources in Yunnan, School of Life Sciences, Yunnan University, Kunming 650500, China; ttoymyy@163.com (T.S.); dxiaotongyn@163.com (X.D.); yuzhangqbl@163.com (Y.Z.)

**Keywords:** nematode-trapping fungi, *Caenorhabditis elegans*, adhesive material, CFEM, pathogenicity

## Abstract

*Arthrobotrys flagrans*, a typical nematode-trapping fungus (NTF) that produces a three-dimensional adhesive network to capture nematodes, has excellent potential for the development of biocontrol agents against both plant and animal parasitic nematodes. Proteins containing the common fungal extracellular membrane (CFEM) domain are important for the nematodes’ trapping by *A. flagrans*. The loss of *AfCFEM1* and *AfCFEM3* resulted in a significant upregulation of proteins associated with fungal pathogenicity, forming a denser adhesive material on the trap surface and ultimately increasing nematode mortality. However, the disruption of *AfCFEM2* led to the opposite result. Furthermore, the deletion of *AfCFEM1-3* not only affected trap morphology, resulting in an increased proportion of irregular traps (i.e., trap cells not fused to the hyphae), but also led to a thinner cell wall of the traps. In addition, the compensatory effects among the CFEM family and other families were demonstrated. This study revealed that the *AfCFEM1-3* genes in *A. flagrans* participated in the formation of traps, adhesive material and cell wall, and pathogenicity, providing new insights into the functions of AfCFEM in the process of nematode trapping by NTF.

## 1. Introduction

Plant and livestock parasitic nematodes cause serious economic losses globally every year [1,2,3]. Synthetic pesticides are common worldwide, but their excessive use is harmful to humans, plants, and agricultural ecosystems, and has led to the emergence and spread of drug resistance [1,4]. Chemical nematicides are banned or restricted, and alternatives to these are urgently needed [1]. A biocontrol agent (BCA) is either a living organism or a natural substance developed based on fungi, bacteria, and other organisms [1]. As natural enemies, nematode trapping fungi (NTF) produce various traps to capture nematodes and finally kill them, making them an ideal source for developing BCAs for managing parasitic nematodes [5]. As one of the typical NTFs, *Arthrobotrys flagrans*, which produces a three-dimensional network, has been proven to be efficient in controlling animal parasitic nematodes [6,7,8]. Furthermore, it was found that *A. flagrans* reduced the number of *Xiphinema index* juveniles in pot cultures of *Ficus carica* and was efficient in capturing *Meloidogyne* spp., colonizing the plant root system, and increasing phosphorus uptake, thereby promoting plant growth [9,10,11,12,13]. Thus, *A. flagrans* has great potential for the development of biocontrol agents against both plant and animal parasitic nematodes.

In the sequence of events involved in NTF infestation of nematodes (recognition and adhesion, penetration, digestion, and growth into host tissues), strong adhesion to the host surface is a prerequisite for penetration of the nematode stage [14,15]. Ultrastructural studies demonstrated that the NTF utilizes an adhesive layer covering the surfaces of the trap to capture nematodes; denaturation of the adhesive layer leads to a significant reduction in nematode capture efficiency [16]. A positive correlation was present between the thickness of the NTF adhesion layer and the number of up-regulated adhesion proteins [17,18,19]. Fungal extracellular membrane proteins of plant- and animal-pathogenic fungi contain the CFEM domain, which was implicated in multiple biological functions in fungi. For example, deleting *PeCFEM5* and *PeCFEM8* in *Penicillium expansum* caused reduced pathogenicity and patulin accumulation [20]. The CFEM domain of Pth11 in *Magnaporthe oryzae* is essential for the development of appressoria and virulence [21]. Three CFEM proteins in *Candida albicans* were shown to participate in hemoglobin-iron acquisition [22]. Evidence suggests that some CFEM-containing proteins are used as cell-surface receptors, signal transducers, or adhesion molecules in host–pathogen interactions, or are involved in virulence, the formation of the inner layer of the cell wall, and heme binding in fungi [20,21,22,23,24,25]. Pathogenic fungi contain more CFEM structural domain-containing proteins than non-pathogenic fungi, suggesting that the CFEM structural domain may play a potentially critical role in fungal virulence [24].

Unsurprisingly, a large number of CFEM-containing proteins (12 in *Drechslerella stenobrocha* and 17 in *A. oligospora*) are also present in NTF, and it is hypothesized that they serve an important role in nematode trapping [26]. However, the specific roles of CFEM domain-containing proteins in nematode trapping by NTF remain to be elucidated. A total of 14 CFEM domain containing genes were differentially expressed in *A. flagrans*, and the expression levels of *AfCFEM1-8* were significantly up-regulated after 18 h of interaction with *C. elegans*. This study was designed to investigate the biological functions of *AfCFEM1-3* in *A. flagrans*, which provides a basis for elucidating the role of CFEM domain-containing genes in nematode trapping by *A. flagrans.*

## 2. Materials and Methods

### 2.1. Organisms and Media

The fungus *A. flagrans* and its knockout mutants, as well as overexpression transformants, were maintained on potato dextrose agar (PDA) plates at 28 °C. Tryptone glucose (TG), tryptone yeast-extract glucose agar (TYGA), potato sucrose tryptone yeast-extract agar (PSTYA), corn meal agar (CMA), Luria–Bertani medium (LB), chlamydospore induction (CI), and WA (1.5% agarose) media were used in this research (Supplementary Information for details). *Caenorhabditis elegans* N2 strain was maintained at 20 °C on nematode growth medium (NGM) plates and fed with concentrated *Escherichia coli* OP50.

### 2.2. Formation of Traps in A. flagrans After Induction by C. elegans

The *A. flagrans* cultured on PDA plates at 28 °C for 4 days was incubated on 100 mL of CI medium in a shaker rotating at 28 °C, 180 rpm for 12 h and then at 90 rpm for 3 days to obtain chlamydospores. Approximately 10^4^ chlamydospores were coated on cellophane-covered CMA plates (9 cm) and left to germinate until mycelium was present throughout the plate. Approximately 1000 *C. elegans* (L2–L4 period) were added to the CMA plates with *A. flagrans*, and the formation of traps was observed and recorded. The experiment was performed in three replicates.

### 2.3. Transcriptome Analysis of A. flagrans Interacted with C. elegans

According to 2.2, at 3, 6, 12, 18, and 24 h of nematode induction, the traps of *A. flagrans* developed into different morphologies. Therefore, the *A. flagrans* and *C. elegans* were collected together after adding approximately 1000 *C. elegans* to each plate for 3, 6, 12, 18, and 24 h, and marked as AC_3, AC_6, AC_12, AC_18, and AC_24, respectively. Samples collected immediately after adding *C. elegans* to *A. flagrans* (*A. flagrans* and *C. elegans* interacted for 0 h) were used as controls. Collected samples were immediately put into liquid nitrogen for freezing before being sent to Shanghai Majorbio Bio-Pharm Technology Co., Ltd. (Shanghai, China) for sequencing, and the transcriptome data were analyzed using the Majorbio cloud platform (https://www.majorbio.com, accessed on 11 June 2023). The experiment was performed in three replicates.

### 2.4. Analysis of Physicochemical Properties and Domains of AfCFEM1-3

Amino acid sequences of three AfCFEM proteins, AfCFEM1 (DFL_009810), AfCFEM2 (DFL_002456), and AfCFEM3 (DFL_009809) were downloaded from the Protein database of the National Center for Biotechnology Information (NCBI). The InterProScan (https://www.ebi.ac.uk/interpro/, accessed on 11 June 2023) and ExPASy ProtParam tool (https://web.expasy.org/protparam/, accessed on 11 June 2023) were used to predict functional domains and identify the physicochemical properties of these three proteins.

### 2.5. Deletion and Overexpression of AfCFEM1-3

Homologous recombination was applied to delete the *AfCFEM1-3*. With the paired primers (Table S1), designed using CE Design (https://crm.vazyme.com/cetool/simple.html (accessed on 11 June 2023), the upstream and downstream fragments of the *AfCFEM1-3* genes and the hygromycin resistance gene (*Hyg*) fragment were amplified using the *A. flagrans* genome and the pCSN44 plasmid as templates, respectively. The *AfCFEM1-3* knockout vectors were constructed by ligating the amplified upstream, downstream, and *Hyg* resistance fragments to the pCE-Zero plasmid (Nanjing Vazyme Biotech Co., Ltd., Nanjing, China). Using *AfCFEM1-3* knockout vectors as a template, the knockout fragments for *AfCFEM1-3* amplified with primer AfCFEM-up-F1-3 and AfCFEM-down-R1-3 (Table S1) were used for protoplast transformation to obtain the knockout mutants as described [27]. The *AfCFEM1-3* deletion mutants were verified by PCR amplification with verification paired primers (F1/R1, F1/R2, F2/R1, and F3/R3) (Table S1).

The *AfGpdP* (glyceraldehyde-3-phosphate dehydrogenase promoter) [27], coding sequences (CDS) of *AfCFEM1-3*, *AfGpdT* (glyceraldehyde-3-phosphate dehydrogenase terminator), and *Hyg* fragments were amplified with specific paired primers (Table S1) and were cloned into the pCE-Zero plasmid to obtain the overexpression vectors of *AfCFEM1-3*. Total RNA of *A. flagrans* that interacted with *C. elegans* for 18 h was extracted with UNlQ-10 Column Total RNA Purification Kit (Sangon Biotech (Shanghai) Co., Ltd., Shanghai, China) and was used as a template for the reverse transcription to cDNA of *AfCFEM1-3*. The overexpression fragments were amplified with primer pCE-Zero-ZH-F/R for protoplast transformation. The total RNA obtained from overexpression transformants, which interacted with *C. elegans* for 18 h, was reverse transcribed to cDNA. The cDNA samples were used as templates to detect the transcription of *AfCFEM1-3* in *AfCFEM1-3* overexpression transformants by RT-qPCR assays. RT-qPCR was carried out with specific paired primers (designed by Primer 5, Table S1) and AceQ Universal SYBR qPCR Master Mix (Vazyme) using a LightCycler 480 Ⅱ (Roche Diagnostics GmbH, Mannheim, Germany), and data were analyzed using LightCycler^®^ 480 SW 1.5.1. The 2^−ΔΔCT^ method was used to quantify relative transcription levels of *AfCFEM1-3* with the *AfGpd* gene as the reference. A melt curve was performed at the end of each reaction to verify PCR product specificity. The experiment was repeated in three replicates for each strain. The transformants with the highest *AfCFEM1-3* gene expression were selected by RT-qPCR analysis for subsequent experiments.

### 2.6. Determination of the Effect of AfCFEM1-3 on Trap Formation and Pathogenicity

L4-stage *C. elegans* worms were prepared as described previously [28]. The WT, Δ*AfCFEM1-3* mutants and *OEAfCFEM1-3* transformants were incubated on WA plates (3.5 cm) at 28 °C for 3 days. The nematode mortality of each plate was calculated after 200–300 L4-stage *C. elegans* were added to the plates for 12, 15, 18, 24, and 48 h. The number of traps was counted at 12 h and 24 h. The proportion of regular and irregular traps was observed at 12 h, and the proportions of single-ring and multiple-ring traps were counted at 24 h. The experiment was performed in three replicates.

### 2.7. Cryo-Scanning Electron Microscope and Transmission Electron Microscope Observation

The WT, Δ*AfCFEM1-3* mutants and *OEAfCFEM1-3* transformants were cultured on WA plates (6 cm) at 28 °C for 4 days, and about 500 *C. elegans* were added to the plates to induce the trap formation of *A. flagrans*. After 15 h, the mycelium and nematodes were collected from each plate and then sent to the Kunming Institute of Botany, Chinese Academy of Sciences to complete sample pre-treatment and cryo-scanning electron microscopy (cryo-SEM) observation. For cryo-SEM, the sample was attached vertically to the sample holder using conductive adhesive. Then, the sample was freeze-fixed with a liquid nitrogen slurry, sublimated at −90 °C for 15 min, and transferred to the cold stage of the scanning electron microscope (−140 °C) under low-temperature and vacuum conditions for observation. The images were obtained using a cryo-scanning electron microscope (Zeiss Sigma 300, Oberkochen, Germany) equipped with a Quorum PP3010T cryo preparation system (East Sussex, UK), operated at 5–7 kV.

The WT and Δ*AfCFEM1-3* mutants were incubated on cellophane-covered WA plates (6 cm) at 28 °C for 4 days, adding about 500 *C. elegans*. The mycelium and nematodes were collected when the *C. elegans* interacted with the mycelium for 18 h. The samples were fixed in 2.5% glutaraldehyde for 12 h and then sent to the Kunming Institute of Zoology, Chinese Academy of Sciences, to complete sample pre-treatment and transmission electron microscopy (TEM) observation. More than 30 trap cells (electron-dense microbodies are present in the cytoplasm) were randomly selected, with 5 randomly selected points per cell, and their cell wall thickness was measured using the Photoshop scale tool. For TEM, the samples were fixed overnight at 4 °C using 2.5% glutaraldehyde in 0.1 M phosphate buffer (pH7.2). Samples were then washed three times with 0.1 M phosphate buffer (pH7.2) for 15 min. Afterward, samples were postfixed with 1% OsO_4_ and 1.5% K_3_[Fe(CN)_6_] for 2 h at 4 °C, then washed with ddH_2_O three times for 7 min, followed by serial ethanol dehydration and acetone transition for 5 min, embedding in SPI Pon 812R resin, polymerization at 60 °C for 48 h. Serial sections of uniform thickness, 800 nm for semithin sections and 60 nm for ultrathin sections, were made using a Leica EM UC7 ultramicrotome (Wetzlar, Germany). Semithin sections were prepared for light microscopy and ultrathin sections for TEM. Semithin sections were stained with toluidine blue. Ultrathin sections were then loaded onto Cu grids and double-stained with 2% uranyl acetate and lead citrate before observation using a JEM 1400 plus transmission electron microscope (JEOL, Tokyo, Japan) at 80 kV.

### 2.8. Determination of the Relative Transcriptional Level of AfCFEM1-8 in ΔAfCFEM1-3 Mutants

The Δ*AfCFEM1-3* mutants were incubated on cellophane-covered CMA plates (9 cm) at 28 °C for 6 days. After 18 h of Δ*AfCFEM1-3* mutants and *C. elegans* interactions, total RNA was extracted and reverse transcribed into cDNA, which was used as a template for RT-qPCR assays. The specific paired primers were designed using Primer 5 and listed in Table S2. The RT-qPCR assays were performed according to the Section 2.5.

### 2.9. Proteomic Analysis of ΔAfCFEM1 and ΔAfCFEM2 Mutants

The chlamydospores of WT, Δ*AfCFEM1*, and Δ*AfCFEM2* mutants were prepared and cultured on cellophane-covered CMA plates (9 cm) at 28 °C for 3 days as described in 2.2. Approximately 1000 *C. elegans* were added to each plate to interact with the WT, Δ*AfCFEM1*, and Δ*AfCFEM2* mutants for 0, 12, and 24 h. Samples of WT, Δ*AfCFEM1* (AP98) and Δ*AfCFEM2* (BP24) mutants that interacted with *C. elegans* were then collected and labeled as WTP_0, WTP_12, WTP_24, AP98_0, AP98_12, AP98_24, BP24_0, BP24_12, and BP24_24. The collected samples were washed three times with PBS buffer, followed by centrifugation at 4 °C and 5000 g for 30 min to remove the supernatant. Subsequently, the samples were immediately frozen in liquid nitrogen and sent to the Shanghai Majorbio Bio-Pharm Technology Co., Ltd. (Shanghai, China) for proteome sequencing analysis. The data were analyzed using the Majorbio cloud platform (https://www.majorbio.com, accessed on 11 June 2023). The experiment was performed in three replicates.

### 2.10. Statistical Analysis

Statistical analysis and graphs creation were performed in GraphPad Prism 9.5.1 (GraphPad Software, San Diego, CA, USA). Significant differences were identified by analyzing the comparison between the control and treated samples using ANOVA with the Dunnett multiple comparisons test. Unless otherwise stated, error bars represent the standard error of the mean of pooled data, and statistical significance is represented by * *p* < 0.05, ** *p* < 0.01, *** *p* < 0.001, and **** *p* < 0.0001

## 3. Results

### 3.1. The Process of Trap Formation of A. flagrans in Response to C. elegans Induction

**Figure 1:**
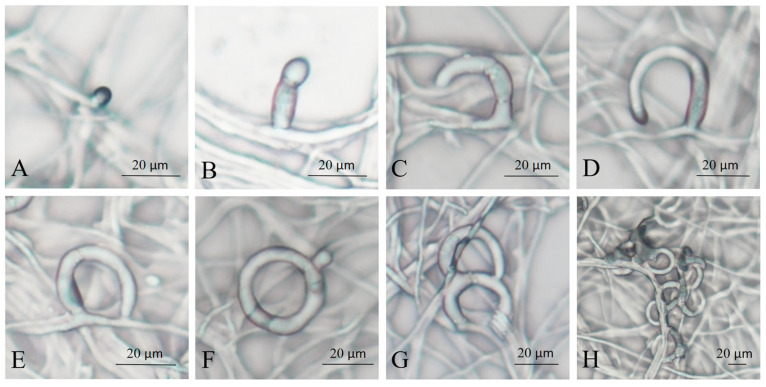
Morphology of *A. flagrans* traps at different times of *C. elegans* induction. (**A**,**B**) 2–3 h; (**C**,**D**) 6–8 h; (**E**,**F**) 8–12 h; (**G**) 12–18 h; (**H**) 24–48 h.

The NTF produces polyketide-derived arthrosporols to inhibit trap formation and releases volatile molecules to attract nematodes [29,30]. When *A. flagrans* detects the highly conserved ascarosides secreted by many soil nematodes, it causes a downregulation of arthrosporol synthesis, thereby initiating trap formation [31,32]. Commonly, traps of *A. flagrans* are only produced after the induction of nematodes, which were considered the most effective inducer for trap production [31,33]. To investigate the process of trap formation of *A. flagrans* after induction by *C. elegans*, regular observations were carried out after *A. flagrans* interaction with the nematodes. The traps of *A. flagrans* began to germinate at 2–3 h (Figure 1A,B) after nematode induction and grew as half rings after 6–8 h (Figure 1C,D); at 8–12 h (Figure 1E,F), most of the traps fused with the mycelium to form a complete closed regular single hyphal loop. After 12–18 h (Figure 1G) of interaction with nematodes, the traps continued to grow to form two or more rings on the base of the single ring. At 24–48 h (Figure 1H) of nematode induction, the traps develop into mature, three-dimensional networks consisting of multiple mycelial rings.

### 3.2. C. elegans Induction Altered Gene Expression in A. flagrans

**Figure 2:**
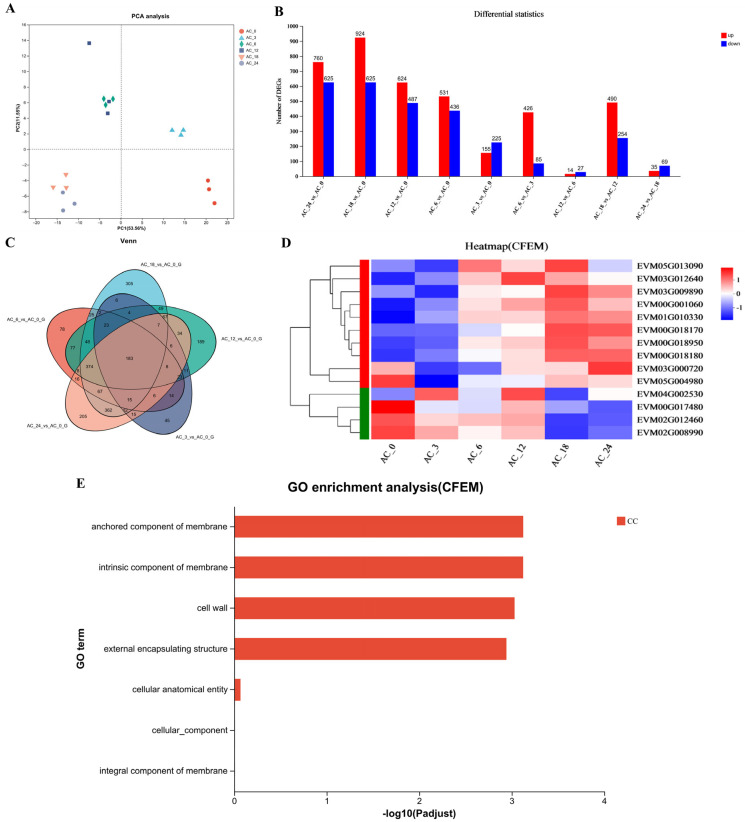
Transcription analysis of *A. flagrans* after *C. elegans* addition. (**A**) Principal component analysis (PCA) plot for *A. flagrans* after induction by *C. elegans* for 0, 3, 6, 12, 18, and 24 h. (**B**) Statistics of differentially expressed genes (DEGs) in *A. flagrans* at 0, 3, 6, 12, 18, and 24 h of *C. elegans* induction. (**C**) Venn analysis of the DEGs in *A. flagrans* induced by *C. elegans* at 0, 3, 6, 12, 18, and 24 h. (**D**) Heatmap of the CFEM protein-related genes expression levels in *A. flagrans* after adding *C. elegans* for 0, 3, 6, 12, 18, and 24 h. (**E**) Gene ontology (GO) enrichment analysis of CFEM protein-related genes.

However, the formation of traps is a highly complicated biological process that may involve numerous genes and pathways [34]. To investigate the changes in gene expression of *A. flagrans* during trap formation after nematode induction for 3, 6, 12, 18, and 24 h compared to 0 h, the transcriptome analysis was performed. Each sample yielded 15.37–40.41 million reads; the quality of the sequence reads was evaluated by a Phred-like quality score, and the Q20, Q30, and GC contents of the clean data were determined (Table S3). More than 90% of the reads matched the reported *A. flagrans* genome [27] after eliminating the *C. elegans* transcripts. Principal component analysis (PCA) indicated high similarity among the three replicates from the same group (Figure 2A). Fold change ≥2 with *p* value < 0.05 was used as a screening criterion during the detection of differentially expressed genes (DEGs). Analysis of the DEGs after addition of *C. elegans* for 3, 6, 12, 18, and 24 h revealed that there were 380 (155 up-regulated and 225 down-regulated), 967 (531 up-regulated and 436 down-regulated), 1111 (624 up-regulated and 487 down-regulated), 1549 (924 up-regulated and 625 down-regulated) and 1385 (760 up-regulated and 625 down-regulated) DEGs were differentially expressed, respectively, compared to 0 h (Figure 2B). Venn analysis of DEGs revealed that 45, 78, 189, 305, and 205 genes were expressed at higher levels after induction of *C. elegans* for 3, 6, 12, 18, and 24 h, respectively. Additionally, 183 DEGs were shared across all selected time points of nematode-*A. flagrans* interactions (Figure 2C).

Furthermore, the Gene Ontology (GO) enrichment analysis and functional enrichment of Kyoto Encyclopedia of Genes and Genomes (KEGG) pathways for DEGs in *A. flagrans* after induction by *C. elegans* for 3, 6, 12, 18, and 24 h, compared with 0 h, were performed. GO enrichment analysis annotates the DEGs to molecular function (MF), cellular component (CC), and biological process (BP) involved. Among them, the MF mainly involve oxidoreductase activity, D-threo-aldose 1-dehydrogenase activity, transporter activity, structural molecule activity, catalytic activity, etc.; the CC are mostly cell wall, membranes, ribosomes, non-membrane-bounded organelle, and nucleolus components; and the BP mainly involve sulfate assimilation, amino acid transport, organic acid transport, peptide metabolic process, translation, carbohydrate metabolic process, glucose metabolic process, etc. (Figure S1). Likewise, the KEGG pathways enriched after nematode induction for more than 3 h included various metabolic processes (sulfur metabolism, tyrosine metabolism, glycolysis/gluconeogenesis, ribosome, etc.) (Figure S1).

In addition, the transcriptomic analysis revealed that the highest number of DEGs appeared 18 h after *C. elegans* induction; therefore, all up-regulated genes at 18 h of nematode induction were ranked according to the fold up-regulation. As a result, most of the top 20 up-regulated genes were related to the toxicity of *A. flagrans* to nematodes, including the Egh16 family (involved in appressorium formation and pathogenesis in pathogenic filamentous fungi), WSC (cell wall integrity and stress response component) domain related genes, and CFEM domain containing genes (Table S4).

As stated earlier, the highest number of DEGs appeared 18 h after *C. elegans* induction. There were 14 CFEM domain-containing genes (Figure 2D) differentially expressed in *A. flagrans*, of which 8 CFEM domain-containing genes, EVM00G018180 (*AfCFEM1*), EVM01G010330 (*AfCFEM2*), EVM00G018170 (*AfCFEM3*), EVM00G018950 (*AfCFEM4*), EVM03G009890 (*AfCFEM5*), EVM00G001060 (*AfCFEM6*), EVM05G013090 (*AfCFEM7*), and EVM03G012640 (*AfCFEM8*) were upregulated 11.56, 8.30, 6.83, 3.03, 2.20, 2.13, 0.98, and 0.85 folds compared to 0 h, respectively, and another 6 CFEM domain-containing genes were down-regulated (Table S5). GO enrichment analysis revealed that the CFEM domain related genes were enriched for cellular components, including cell wall and membrane components (Figure 2E). Combining GO functional annotation, Swiss-Prot annotation information, and relevant reports, we inferred that the CFEM domain-containing genes in *A. flagrans* might be involved in forming cell membranes, cell walls, and cell wall adhesion proteins, or encode proteins anchored to cell membranes. Therefore, three genes (*AfCFEM1*, *AfCFEM2*, *AfCFEM3*) with the highest up-regulation of transcript levels at 18 h of interaction with *C. elegans* were selected for subsequent functional studies.

The CFEM domain is unique to fungi but not present in all fungi [24,35]. The three proteins’ physicochemical properties and functional domains, AfCFEM1-3, were analyzed. The results showed that the AfCFEM1 protein contains 130 amino acids, has a molecular weight of 13.57 KDa, and an isoelectric point of pI of 8.08 (Figure S2); the AfCFEM2 protein contains 208 amino acids, has a molecular weight of 20.59 KDa, and an isoelectric point of pI of 3.76 (Figure S2); the AfCFEM3 protein contains 614 amino acids, has a molecular weight of 64.37 KDa, and an isoelectric point of pI of 3.28 (Figure S2); AfCFEM1-3 proteins all contain the CFEM domain.

### 3.3. AfCFEM1-3 Play a Vital Role in the Trap’s Morphology and Pathogenicity of A. flagrans

With a view to exploring the role of *AfCFEM1-3* in *A. flagrans*, the *AfCFEM1-3* were disrupted and overexpressed, and the Δ*AfCFEM1-3* mutants and *OEAfCFEM1-3* transformants were obtained and verified by PCR amplification (Figure S3) and RT-qPCR (Figure S4), respectively.

**Figure 3:**
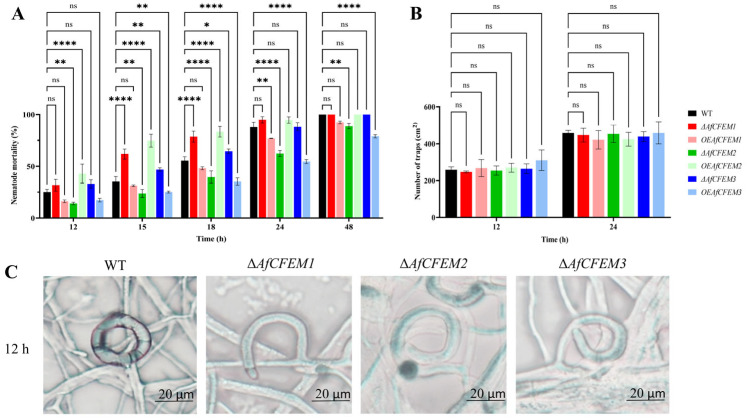
The nematode mortality, number, and morphology of traps in WT, Δ*AfCFEM1-3* mutants, and *OEAfCFEM1-3* transformants during the interaction with *C. elegans*. (**A**) The nematode mortality of the WT, Δ*AfCFEM1-3* mutants, and *OEAfCFEM1-3* transformants at 12, 15, 18, 24, and 48 h after adding *C. elegans*. (**B**) The number of traps of WT, Δ*AfCFEM1-3* mutants, and *OEAfCFEM1-3* transformants per cm^2^ at 12 and 24 h after *C. elegans* addition. (**C**) The morphology of traps in WT and Δ*AfCFEM1-3* mutants after adding *C. elegans* for 12 h. (ns *p* > 0.05, * *p* < 0.05, ** *p* < 0.01, and **** *p* < 0.0001).

After *C. elegans* addition for 12, 15, 18, 24, and 48 h, the nematode mortality results of WT (25.06, 35.41, 55.57, 88.06, and 100%, respectively), Δ*AfCFEM1* mutants (31.72, 61.82, 78.73, 94.87, and 100%, respectively) and *OEAfCFEM1* transformants (16.28, 31.27, 48.11, 77.00 and 92.37%, respectively) were obtained (Figure 3A), the results showed that the loss of *AfCFEM1* caused increased nematode mortality at 12, 15, 18 and 24 h, and was significantly different at 15 and 18 h. For *AfCFEM2*, the deletion of *AfCFEM2* leads to a significant reduction in nematode mortality to 14.10, 23.74, 39.66, 62.38, and 88.88% at 12, 15, 18, 24, and 48 h, respectively (Figure 3A). In contrast, the nematode mortality of the *OEAfCFEM2* transformant was 42.91, 74.76, 83.47, 94.68, and 100%, respectively (Figure 3A). The overexpression of *AfCFEM2* caused a significant increase in nematode lethality at 12, 15, and 18 h. As for *AfCFEM3*, the nematode mortality of Δ*AfCFEM3* mutants was 32.96, 46.96, 64.49, 88.29, and 100% after nematode addition for 12, 15, 18, 24, and 48 h, respectively, in which the nematode mortality was significantly higher than that of WT at 12, 15, and 18 h (Figure 3A). Whereas the overexpression of the *AfCFEM3* gene significantly reduced nematode mortality to 17.38, 25.10, 35.35, 54.61, and 79.02% at 12, 15, 18, 24, and 48 h, respectively (Figure 3A). It can be noticed that both the knockout and overexpression of the *AfCFEM1-3* resulted in changes in nematode mortality. Interestingly, there was no significant difference among the number of traps produced by WT, Δ*AfCFEM1-3* mutants, and *OEAfCFEM1-3* transformants at 12 and 24 h after nematode addition (Figure 3B).

It is noteworthy that *AfCFEM1-3* has an important impact on trap morphology. After 12 h of nematode addition, most of the traps in the WT would fuse with the hyphae to form a closed loop (98.33%) (Figure 3C). Still, some traps in Δ*AfCFEM1-3* mutants were not fused with the hyphae, resulting in traps twisted at different angles (Figure 3C). Thus, the proportion of irregular traps significantly increased to 28.12, 27.28, and 28.82%, respectively.

### 3.4. AfCFEM1-3 Associated with the Cell Wall Formation and Adhesive Proteins in Traps of A. flagrans

**Figure 4:**
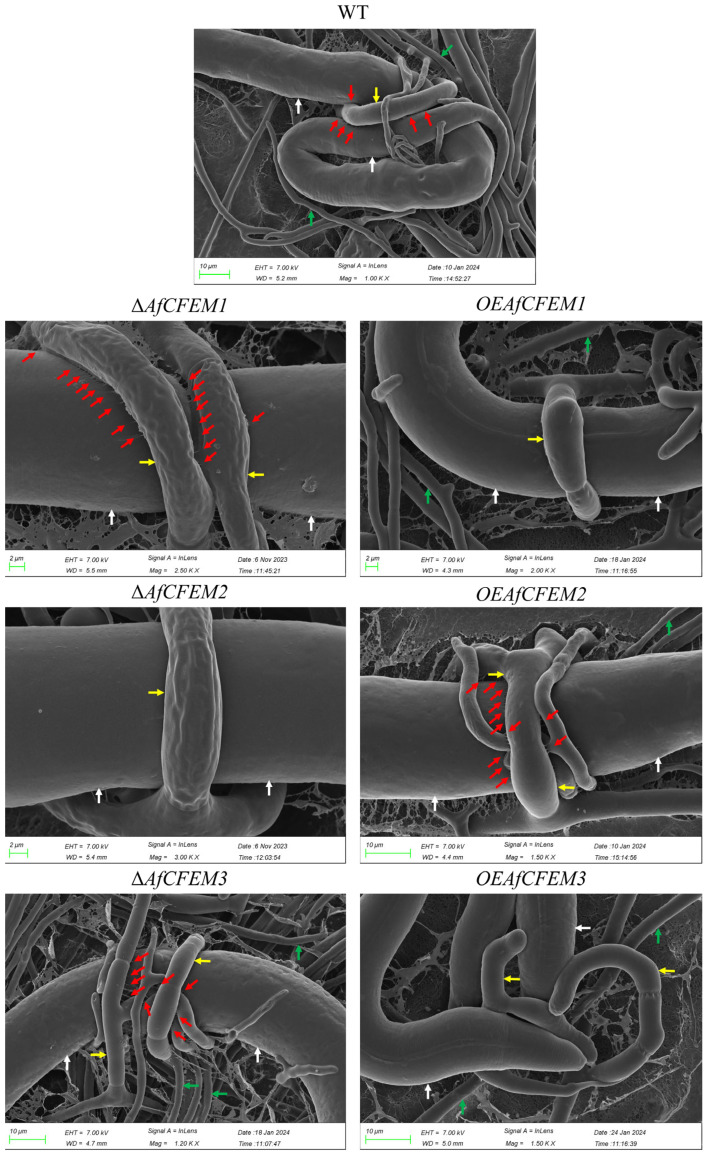
Partial representative cryo-scanning electron microscope images of the adhesive material on the trap–nematode contact surface of traps in WT, Δ*AfCFEM1-3* mutants, and *OEAfCFEM1-3* transformants after the addition of *C. elegans* for 15 h. Red arrow: adhesive material; white arrow: nematode (*C. elegans*); green arrow: mycelium of *A. flagrans;* yellow arrow: trap of *A. flagrans.*

**Figure 5:**
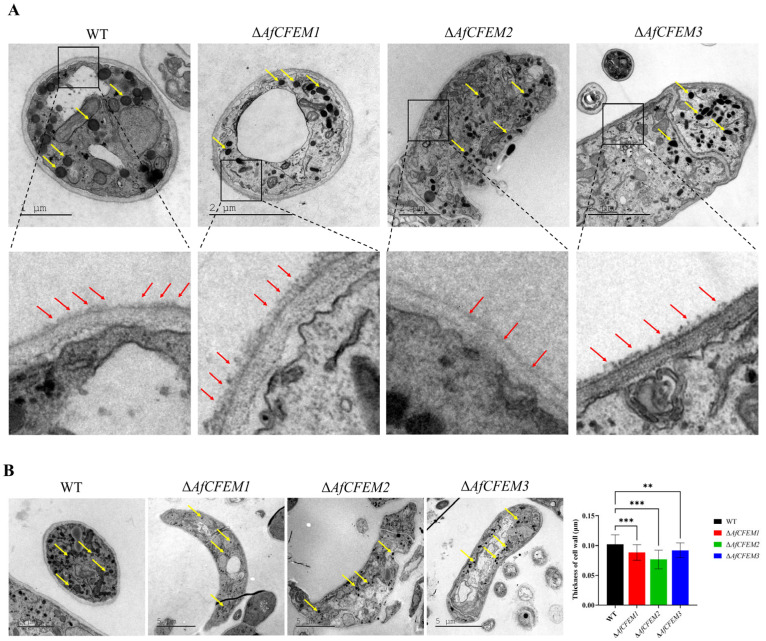
The transmission electron microscope observation on traps of WT and Δ*AfCFEM1-3* mutants. (**A**) The flocculent material (red arrow) presented on the outer surface of the cell walls of the WT and Δ*AfCFEM1-3* mutants traps after 18 h of *C. elegans* addition. Yellow arrow: electron-dense microbodies. (**B**) Partial representative images and cell wall thickness of traps of WT and Δ*AfCFEM1-3* mutants. (** *p* < 0.01, *** *p* < 0.001).

The cryo-SEM results showed that during interactions with *C. elegans* for 15 h, the adhesive material present on the trap–nematode contact trap surface of Δ*AfCFEM1* and Δ*AfCFEM3* mutants (Figure 4) was denser than that of WT. In contrast, it was barely observed in the *OEAfCFEM1* and *OEAfCFEM3* transformants (Figure 4). However, little adhesive material was observed on the trap surface of the Δ*AfCFEM2* mutants, and there was an increasing amount of adhesive substance on the trap surface of the *OEAfCFEM2* transformants (Figure 4). The electron-dense microbodies in the cytoplasm are features shared by the morphologically diverse trap cells produced by NTF, distinguishing them from hyphal cells [34]. Therefore, trap cells in TEM images were observed using electron-dense microbodies in the cytoplasm as a criterion for identification. The TEM results showed that flocculent material was present on the outer surface of the cell walls of the WT, Δ*AfCFEM1*, and Δ*AfCFEM3* strains traps after 18 h of nematode addition, but was barely observed in the cell walls of the Δ*AfCFEM2* mutants (Figure 5A). Moreover, the knockout of the *AfCFEM1-3* leads to a significant reduction in the cell wall thickness of the *A. flagrans* traps (Figure 5B).

### 3.5. The Compensating Effects Exist Among AfCFEM1-8 of A. flagrans

**Figure 6:**
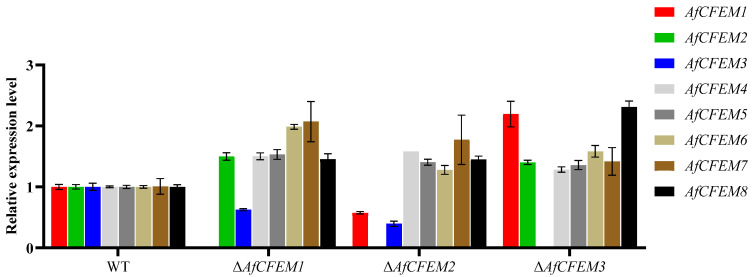
The relative expression levels of *AfCFEM1-8* in WT and Δ*AfCFEM1-3* mutants after interacting with *C. elegans* for 18 h.

In *Candida albicans*, the overexpression of genes coding for other proteins having adhesive properties compensates for the loss of the genes encoding CFEM domain-containing proteins [36]. The results revealed that in the Δ*AfCFEM1* mutants, the relative expression levels of the other 6 *AfCFEM* were up-regulated compared to the WT, except for the down-regulated expression of *AfCFEM3* (Figure 6). In addition, the relative expression levels of *AfCFEM1* and *AfCFEM3* were down-regulated, and the *AfCFEM4-8* were up-regulated in Δ*AfCFEM2* mutants (Figure 6). For Δ*AfCFEM3* mutants, the relative expression levels of all other *AfCFEM* genes were up-regulated (Figure 6).

### 3.6. AfCFEM1 and AfCFEM2 Affected the Protein Expression in A. flagrans

Proteomics is one of the most important methods for investigating the function of genes. The proteomic statistics and the sample correlation heatmap (Figure S5) show that the proteomic data are of high quality and have good reproducibility between replicates, respectively. Fold change ≥ 1 with *p* value < 0.05 was used as a screening criterion during the detection of differentially expressed proteins (DEPs). The DEPs were statistically analyzed (Figure S6), and we focused on the adhesion-related DEPs that were up-regulated in Δ*AfCFEM1* mutants and down-regulated in Δ*AfCFEM2* mutants, for which the GO and KEGG annotations for the DEPs are shown in Figure S6. A further screening of virulence and adhesion proteins from the above DEPs revealed that a subtilisin-like serine protease (EVM00G001030), an aspartic protease (EVM01G001350), and a malate synthase (EVM01G004180) were up-regulated at Δ*AfCFEM1* mutants at 12 h of interactions with nematodes (Table S6). Moreover, two proteins (EVM01G009790 and EVM02G001720) containing lectin domains (associated with adhesion), two subtilisin-like serine proteases (EVM00G001030 and EVM03G007260), and two aspartic acid proteases (EVM01G001350 and EVM02G016290) were up-regulated after Δ*AfCFEM1* mutants interacted with *C. elegans* for 24 h (Table S6). In contrast, an Egh16 virulence protein (EVM02G005160) and a lipase-related protein (EVM00G010160) were down-regulated at Δ*AfCFEM2* mutants that interacted with nematodes for 12 h and 24 h, respectively (Table S6). Additionally, many of the up-regulated virulence and adhesion DEPs screened in Δ*AfCFEM1* mutants were annotated to physiological processes, including autophagy, MAPK signaling pathway, lysine biosynthesis, nitrogen metabolism, glycolysis/gluconeogenesis, starch and sucrose metabolism, and citrate cycle (Table S7). The downregulated virulence and adhesion DEPs of Δ*AfCFEM2* mutants also annotated autophagy and MAPK signaling pathways (Table S7). In addition, AfCFEM4 protein expression was up-regulated in both Δ*AfCFEM1* and Δ*AfCFEM2* mutants.

## 4. Discussion

NTF does not spontaneously produce or produce a small number of traps. Traps can be induced in response to various inducers such as nematode extract, amino acids, small peptides, abscisic acid, ascarosides, and other substances [34]. However, living nematodes are considered the most effective inducer for trap production [33]. Here, it was observed that *C. elegans* induced traps of *A. flagrans* sprouted from hyphae and gradually developed into closed single-loop traps, eventually maturing into three-dimensional networks with multiple mycelial rings. Many genes in *A. flagrans* are differentially expressed during nematode-induced trap formation. The top 20 genes up-regulated after nematode induction for 18 h contained four Egh16 family genes, WSC domain-related genes, and CFEM domain-related genes, suggesting that they are critical in trap formation. It was also shown that Egh16 family genes are important for host attachment devices (traps and appressorium) development of fungi [37,38]. In contrast, WSC domain-containing proteins were found to function as cell wall sensors and were involved in fungal adhesion [39,40,41,42]. In addition, it was demonstrated that the expression level of the WSC domain was significantly higher in traps than in the mycelium of NTF [43]. Moreover, CFEM domain containing proteins were demonstrated to participate in biofilm formation and cell wall biogenesis [24,44,45]. The transcriptomes indicated that the CFEM domain containing proteins participated in the trap formation processes of NTF *Drechslerella dactyloides* [46]. The protein encoded by *AfCFEM5* (EVM03G009890) is a homolog of the adhesive protein AoMAD1 of *A*. *oligospora* with 83.3% homology. Therefore, the CFEM containing genes in *A. flagrans* might be associated with the formation of cell membranes and cell wall adhesion proteins.

Transcriptomic analysis showed proteins containing the CFEM domain serve an important role in nematode trapping by *A. flagrans*. Pathogenic fungi contain more fungal-unique CFEM domains than non-pathogenic fungi, indicating that the amount of CFEM is positively correlated with fungal pathogenicity [24]. Target deletion of *AfCFEM1-3* leads to alterations in nematode mortality, trap morphology, adhesive materials on the surface of traps, and cell wall thickness of *A. flagrans*. The knockout of *AfCFEM1* and *AfCFEM3* resulted in an increase in the adhesive material on the surface of the trap cells, contributing to the rise in nematode mortality. Whereas the loss of *AfCFEM2* caused a reduction in adhesive material on the trap’s surface, which decreased the nematode mortality. Therefore, the CFEM domain-containing gene, *AfCFEM1-3*, of *A. flagrans* mediate virulence by participating in the adhesive material formation on the cell surface of the trap, which eventually leads to changes in nematode mortality.

Trap cell wall thickness was significantly thinned due to the loss of *AfCFEM1-3*, indicating that *AfCFEM1-3* is involved in trap cell wall synthesis of *A. flagrans*. This result is consistent with Mrsa et al., who identified the *CCW14* gene (containing the CFEM domain) as a covalently linked cell wall protein of *Saccharomyces cerevisiae* [24,47]. Moreover, the expression level of the CFEM domain containing gene *CgCFEM1* in appressoria of *Colletotrichum gloeosporioides* was two hundred times more than that in mycelia and conidia [48]. Evidently, the CFEM domain is of extraordinary significance for pathogenic fungi that possess adhesion devices, such as traps and appressoria.

NTF secret extracellular enzymes, including subtilisin-like serine proteases, chitinase, and lipase, degrade the nematode cuticle and destroy the nematode epidermis, which helps digest the nematodes [49,50]. Malate synthase, a key enzyme in the glyoxylate cycle, is required for the virulence of microbial pathogens [51]. It has been reported that the malate synthase is important for trap formation and pathogenicity of *A. oligospora* [52]. Moreover, aspartic proteases have been reported to be implicated in the mycoparasitic process [51]. Proteome data show that subtilisin-like serine protease (EVM00G001030 and EVM03G007260) and malate synthase (EVM01G004180) were up-regulated in Δ*AfCFEM1* mutants after 12 h and 24 h of interactions with nematodes. In contrast, an Egh16 virulence protein (EVM02G005160) and a lipase-related protein (EVM00G010160) were down-regulated in the Δ*AfCFEM2* mutants after interacting with nematodes for 12 h and 24 h, respectively. This demonstrates that the Δ*AfCFEM1* mutants are more active than the WT in the processes of nematode adhesion, penetration, and digestion, while the opposite is true for the Δ*AfCFEM2* mutants. Additionally, numerous autophagy-related genes in nematode trapping fungi are involved in the positive regulation of trap formation and pathogenicity [53,54,55]. In contrast, many of the upregulated pathogenicity and adhesion DEPs identified in the Δ*AfCFEM1* mutants were annotated as being associated with physiological processes, including autophagy, MAPK signaling pathway, lysine biosynthesis, nitrogen metabolism, glycolysis/gluconeogenesis, starch and sucrose metabolism, and citrate cycle. The virulence and adhesion DEPs downregulated in the Δ*AfCFEM2* mutants were also annotated in the autophagy and MAPK signaling pathways. Therefore, the proteomic results partially explain the higher and lower nematode mortality of Δ*AfCFEM1* and Δ*AfCFEM2* mutants than that of the WT at the protein expression level, respectively. Interestingly, the deletion of *AfCFEM1* caused the two proteins (EVM01G009790 and EVM02G001720) containing lectin domains (associated with adhesion) to be up-regulated. The cryo-SEM also showed more adhesive materials on the cell wall surface of traps of the Δ*AfCFEM1* mutant than in WT. This result and another reported study [56] demonstrated that genetic damage caused by the deletion of CFEM family genes was compensated for by significantly increasing the expression levels of other family members. Furthermore, detecting the relative expression of other *AfCEFM* members in the Δ*AfCFEM1-3* mutants showed that the compensatory mechanism also exists among CFEM family members in *A. flagrans*. This suggests that compensation mechanisms exist between CFEM families and other families and among members of CFEM families in *A. flagrans.*

Not only were nematode mortality and adhesive material on the surface of trap cells affected by the loss of *AfCFEM1-3*, but also the morphology of the traps was affected. The knockout of *AfCFEM1-3* all caused an increased proportion of irregular traps that failed to form closed loops. The three-dimensional adhesive networks of *A. flagrans* and *A. oligospora* result from multiple cell fusions (anastomoses) [57]. The study of *A. oligospora* trap formation revealed that trap cells grow perpendicular to the parental hyphae, subsequently bend, and fuse with the small pegs that develop on the hyphae toward the trap cells, eventually forming a closed ring [58]. Intercellular communication is required for loop closure during the formation of traps in *A. flagrans* and *A. oligospora* [59]. The loss of the hyphal anastomosis gene *SofT* in *A. flagrans* disrupted the ring closure step, leading to spiral hyphae [57]. Therefore, the CFEM domain-containing genes *AfCFEM1-3* are critical for cellular communication during trap formation.

## 5. Conclusions

The present study reports that *AfCFEM1-3* were essential for producing adhesive materials, trap morphology, cell wall biogenesis, and pathogenicity of *A. flagrans*. The loss of *AfCFEM1-3* caused changes in nematode mortality, trap surface adhesive substances, and trap cell wall thickness. Moreover, the absence of *AfCFEM1-3* led to an increased proportion of irregular trap formation. By combining the results of RT-qPCR and proteomic analysis, the compensating effects among the *CFEM* family and between other families were demonstrated in *A. flagrans*. Taken together, this study elucidated functions of *AfCFEM1-3*, which contribute to a deeper understanding of the biological functions of adhesive proteins in nematode killing by *A. flagrans*.

## Data Availability

The published paper and the associated Supplementary Files include all data generated or analyzed during this study. The RNA-seq data presented here are associated with NCBI BioProject PRJNA1307640. The mass spectrometry proteomics data have been publicly released via the iPox partner repository with the identifier PDX:067507.

## Supplementary Materials

The following supporting information can be downloaded at: https://www.mdpi.com/article/10.3390/microorganisms13092001/s1. Supplementary Figure S1: Gene Ontology (GO) enrichment analysis and Kyoto Encyclopedia of Genes and Genomes (KEGG) enrichment of DEGs in *A. flagrans* at 3, 6, 12, 18, and 24 h of *C. elegans* induction compared with 0 h. Supplementary Figure S2: Prediction of conserved domains of AfCFEM1-3 proteins. Supplementary Figure S3: Verification of the Δ*AfCFEM1-3* mutants by PCR amplification using F1/R2, F2/R1, and F3/R3 primer pairs. ‘+’ and ‘−’ represent positive control and negative control, respectively. Supplementary Figure S4: Verification of the *OEAfCFEM1-3* transformants by RT-qPCR analysis using specific primer pairs. Supplementary Figure S5: Basic information of protein identification. Supplementary Figure S6: Proteomic analysis of Δ*AfCFEM1* and Δ*AfCFEM2* mutants at 0, 12, and 24 h of *C. elegans* induction compared to WT. Supplementary Table S1: List of primers used in the knockout and overexpression of *AfCFEM1-3.* Supplementary Table S2: List of primers used in RT-qPCR to determine the relative transcriptional level of *AfCFEM1-8* genes in Δ*AfCFEM1-3* mutants. Supplementary Table S3: Statistics of reads, phred-like quality scores, and GC content for the *A. flagrans* induced by *C. elegans* for different time points. Supplementary Table S4: The top 20 up-regulated genes in *A. flagrans* at 18 h of interaction with *C. elegans*. Supplementary Table S5: Differential expression data of 14 CFEM protein-related genes in *A. flagrans* after 18 h of interaction with *C. elegans* compared with 0 h. Supplementary Table S6: Differentially expressed virulence and adhesion proteins of knockout strains Δ*AfCFEM1* and Δ*AfCFEM2*. Supplementary Table S7: Pathways annotation of the virulence and adhesion-associated DEPs in Δ*AfCFEM1* and Δ*AfCFEM2* mutants compared to WT.
