# Supplementary material for: Functions of the Three Common Fungal Extracellular Membrane (CFEM) Domain-Containing Genes of Arthrobotrys flagrans in the Process of Nematode Trapping
Source: Microorganisms. 2025 Aug 27;13(9):2001. doi: 10.3390/microorganisms13092001 (PMC12471864; doi:10.3390/microorganisms13092001)
Supplement: Supplementary file 1 [file microorganisms-13-02001-s001.zip › microorganisms-3804554-supplementary.pdf]

Supplementary materials for

Functions of the three common fungal extracellular membrane (CFEM) domain-containing genes of *Arthrobotrys flagrans* in the process of nematode trapping

Tingting Shi†, Xiaotong Deng†, Yu Zhang, Guohong Li*

State Key Laboratory for Conservation and Utilization of Bio-Resources in Yunnan, School of Life Sciences, Yunnan University, Kunming 650500, PR China

* Corresponding author E-mail: ligh@ynu.edu.cn (G.L.)

† These authors contributed equally to this work

Media

PDA, boil 200 g of potatoes for 30 minutes and filter to get the filtrate, 20 g of glucose and 15 g of agar and refill with distilled water to 1 L. **TG**, 10 g of tryptone, 10 g of glucose and 15 g of agar per 1 L of distilled water. **TYGA**, 10 g of tryptone, 10 g of glucose, 5 g of yeast extract, 5 g of syrup and 15 g of agar per 1 L of distilled water. **PSYTA**, boil 200 g of potatoes for 30 minutes and filter to get the filtrate, 206 g of sucrose, 0.5 g of yeast extract, 0.5 g of tryptone and 15 g of agar per 1 L of distilled water. **CMA**, boil 20 g of corn and filter to get the filtrate, 15 g of agar and refill with distilled water to 1 L. **LB**, 5 g of yeast extract, 10 g of tryptone, 10 g of NaCl and 15 g of agar per 1 L of distilled water. Chlamydospore induction (**CI**) medium, 10 g of sucrose, 0.1 g of $NaNO_3$, 0.5 g of $MgSO_4$, 0.01 g of $FeSO_4$, 0.5 g of KCl and 1.0 g of K_2HPO_4 per 1 L of distilled water. **WA**, 15 g of agar in 1 L of distilled water.

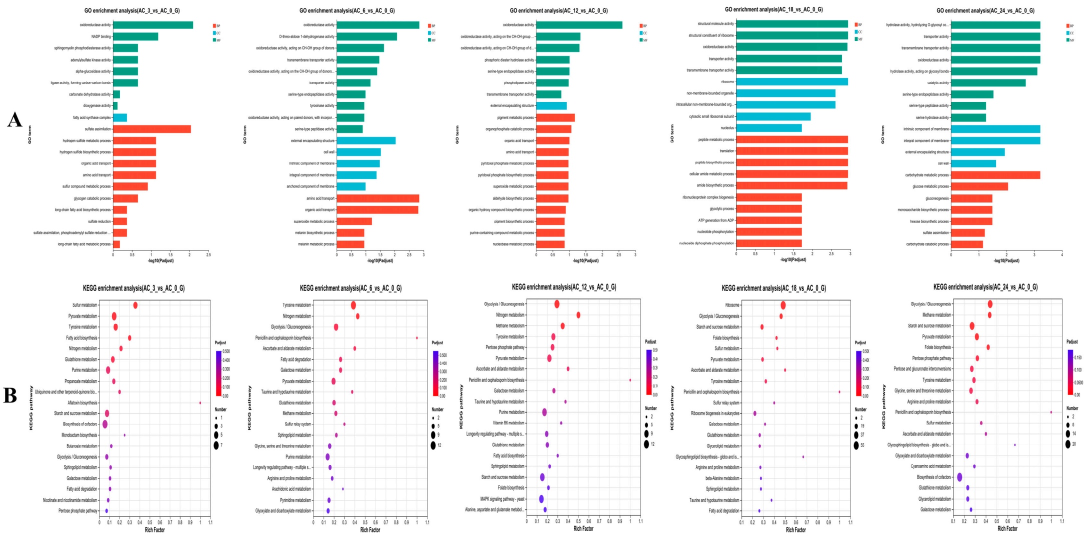

Supplementary Figure S1. Gene Ontology (GO) enrichment analysis and Kyoto Encyclopedia of Genes and Genomes (KEGG) enrichment of DEGs in *A. flagrans* at 3, 6, 12, 18, and 24 h of *C. elegans* induction compared with 0 h. (**A**) GO enrichment analysis. (**B**) KEGG enrichment analysis.

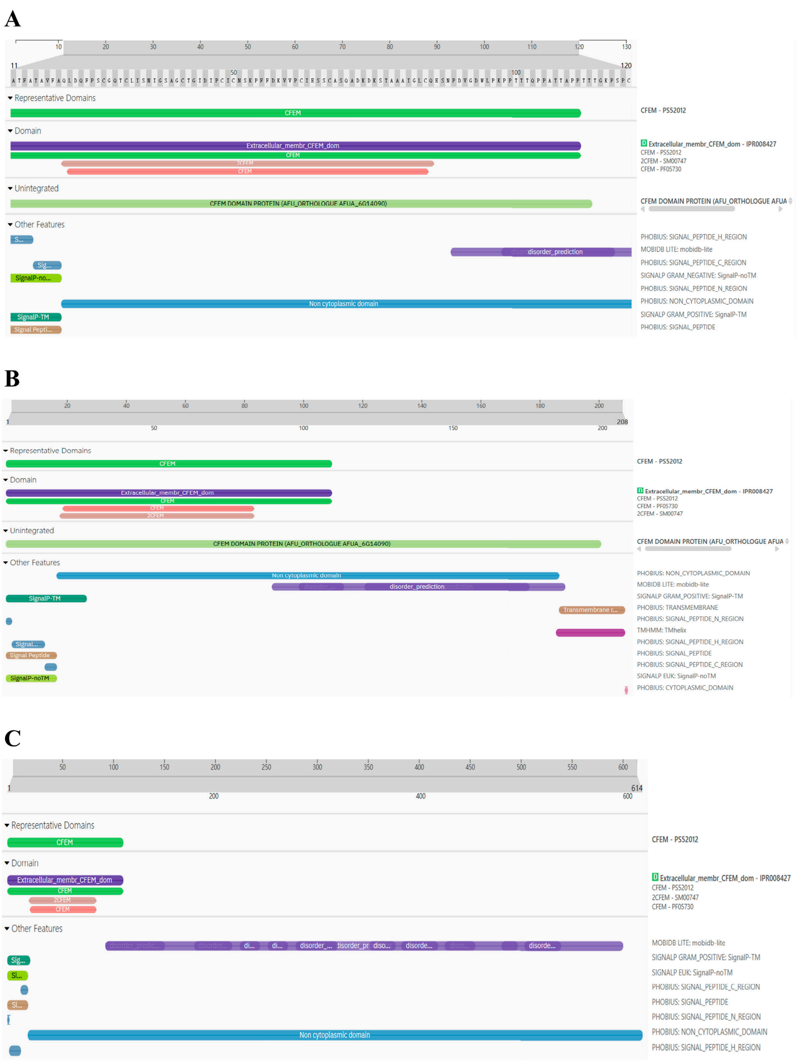

Supplementary Figure S2. Prediction of conserved domains of AfCFEM1-3 proteins. (**A**) AfCFEM1. (**B**) AfCFEM2. (**C**) AfCFEM3.

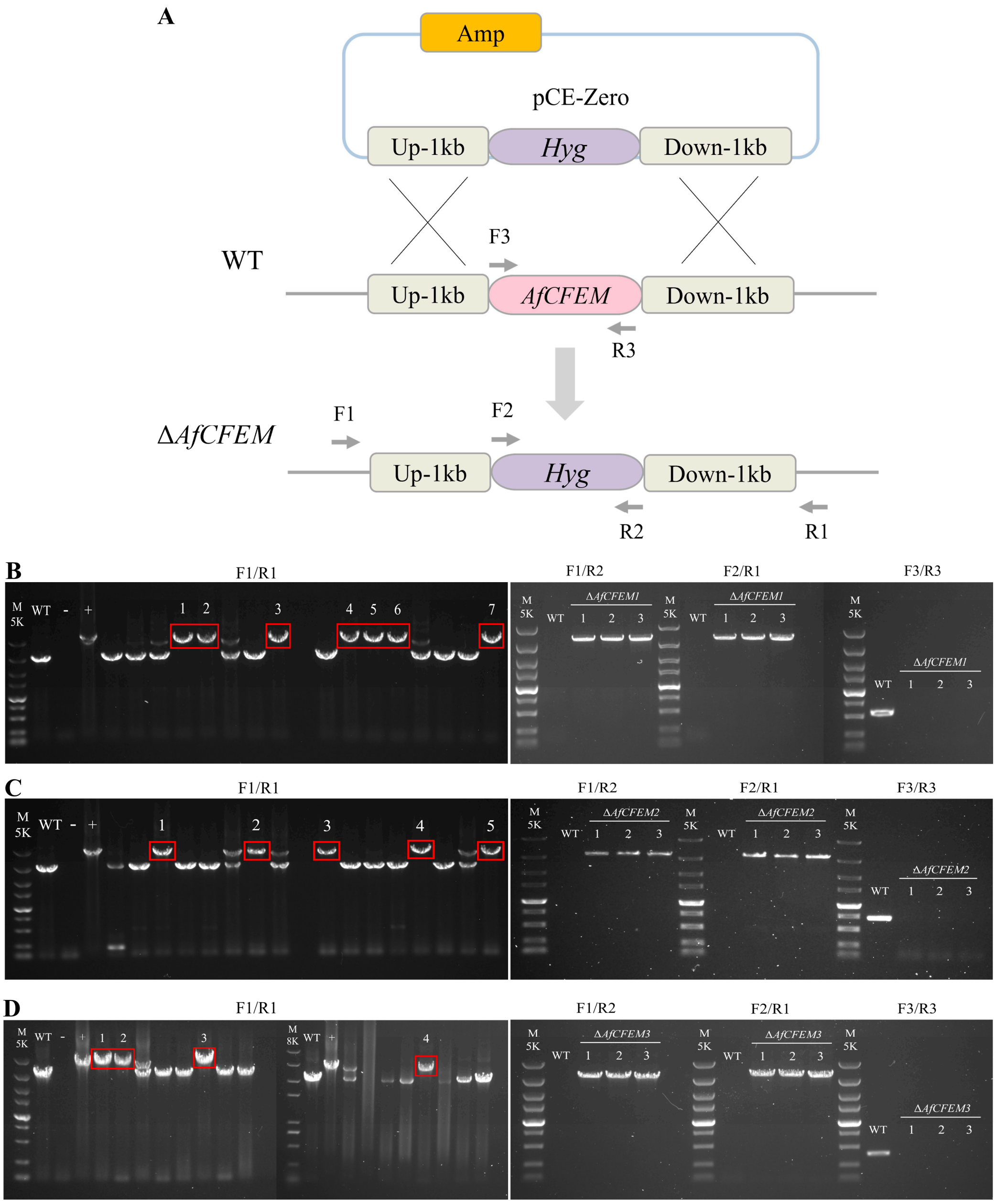

Supplementary Figure S3. Verification of the Δ*AfCFEM1-3* mutants by PCR amplification using F1/R2, F2/R1, and F3/R3 primer pairs. '+' and '-' represent positive control and negative control, respectively. (**A**) Diagrammatic sketch of homologous recombination of *AfCFEM*. (**B**) Verification of the Δ*AfCFEM1* mutants. (**C**) Verification of the Δ*AfCFEM2* mutants. (**D**) Verification of the Δ*AfCFEM3* mutants.

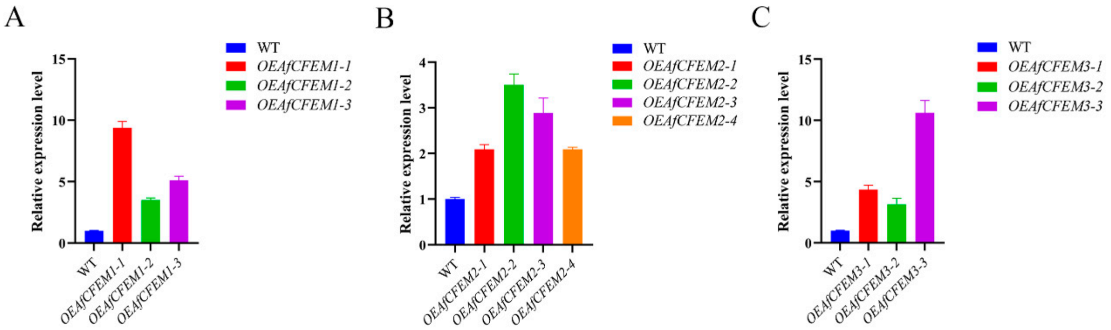

Supplementary Figure S4. Verification of the *OEAfCFEM1-3* transformants by RT-qPCR analysis using specific primer pairs. (**A**) Relative expression level of *AfCFEM1* genes in overexpressed transformants. (**B**) Relative expression level of *AfCFEM2* genes in overexpressed transformants. (**C**) Relative expression level of *AfCFEM3* genes in overexpressed transformants.

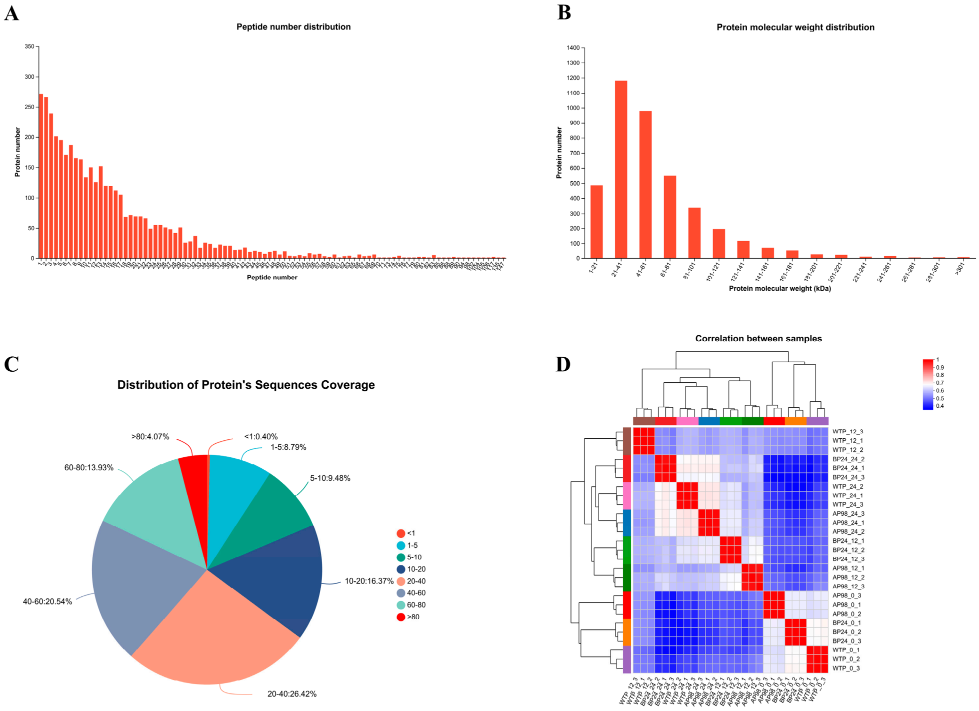

Supplementary Figure S5. Basic information on protein identification. (**A**) Diagram of peptide length distribution in proteomic sequencing. The abscissa represents peptide length, and the ordinate represents peptide quantity. (**B**) Distribution map of protein molecular weight. The abscissa represents the protein molecular weight distribution, and the ordinate represents the protein quantity. (**C**) Protein coverage distribution map. (**D**) Sample correlation heat map.

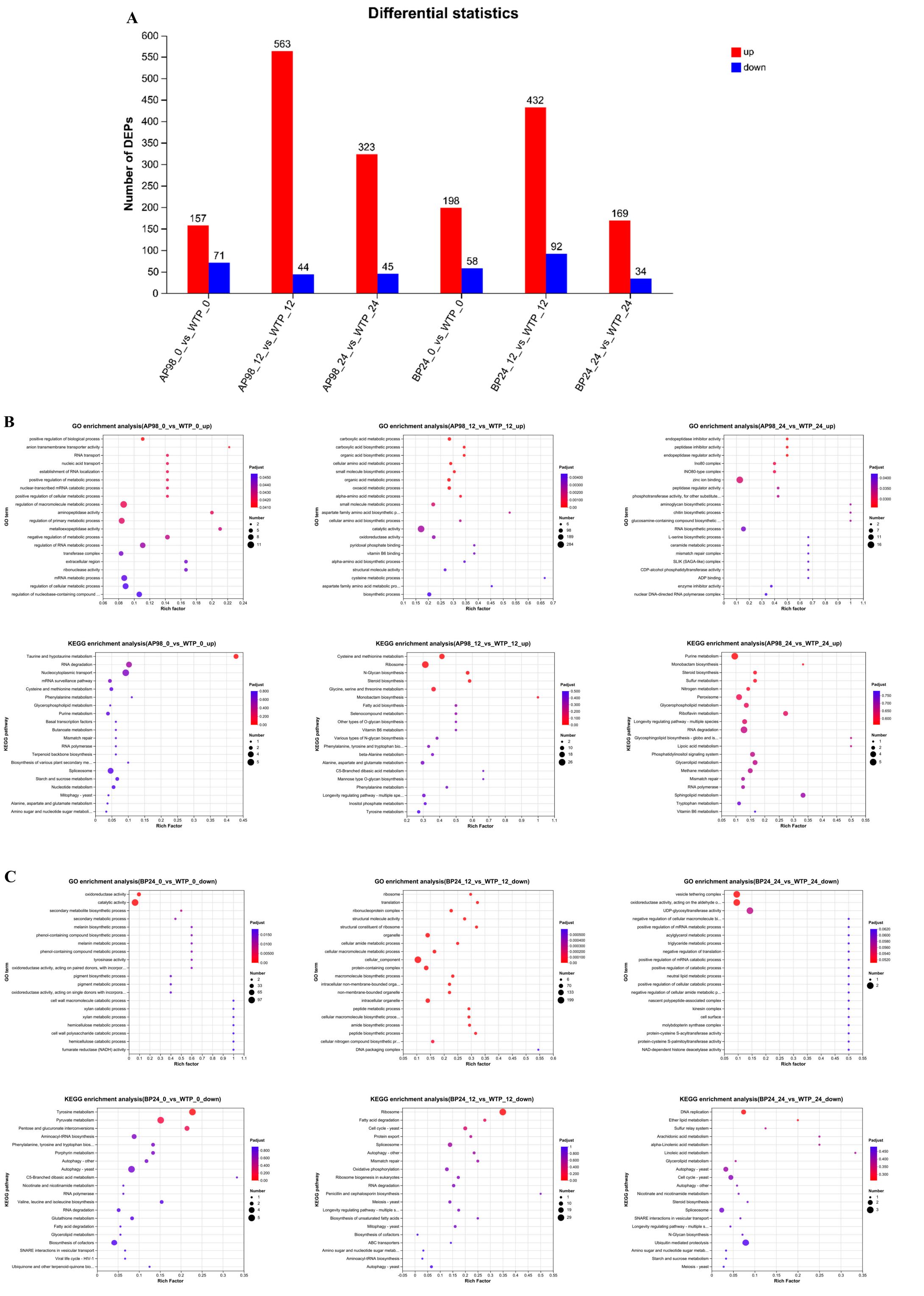

Supplementary Figure S6. Proteomic analysis of Δ*AfCFEM1* and Δ*AfCFEM2* mutants at 0, 12, and 24 hours of *C. elegans* induction compared to WT. **(A)** Statistical diagram of differential proteins. (**B**) GO enrichment analysis and KEGG enrichment analysis result of the up-regulated differential proteins of Δ*AfCFEM1* mutants during 0, 12 and 24 h interaction with *C. elegans*. (**C**) GO enrichment analysis and KEGG enrichment analysis result of the up-regulated differential proteins of Δ*AfCFEM2* mutants during 0, 12 and 24 h interaction with *C. elegans*.

Supplementary Table S1. List of primers used in the knockout and overexpression of *AfCFEM* 1-3.

Primers	Sequence (5′-3′)	Description
AfCFEM1-up-F (F1-1)	TTCGGATCTTCCAGAGTGTAAGCTTTCTTAGAGCGCCTC	Amplify the upstream fragment (1222 bp) of *AfCFEM1* gene; F1-1, verify the *AfCFEM1* deletion mutants
AfCFEM1-up-R	TCTGTTTGAATGTAAGTTGGTTTTGAAAATG	
AfCFEM1-down-F	ATTTCGACAATAGTCGGGCCA	Amplify the downstream fragment (1193 bp) of *AfCFEM1* gene; R1-1, verify the *AfCFEM1* deletion mutants
AfCFEM1-down-R (R1-1)	CAACTGCCGTTCGACTGCGGCGGAACCTAGACTT	
AfCFEM1-hyg-F (F2-1)	CCAACTTACATTCAAACAGAAGATGATATTGAAGGAGCATTT	Amplify the *Hyg* resistance fragments (2121 bp) for *AfCFEM1* gene deletion, verify the *AfCFEM1* deletion mutants
AfCFEM1-hyg-R (R2-1)	GGCCCGACTATTGTCGAAATAAAGAAGGATTACCTCTAAACAAGTGTACC	
AfCFEM2-up-F (F1-2)	TTCGGATCTTCCAGAAAGTTGAAGCGACTTGAGATTTCG	Amplify the upstream fragment (1110 bp) of *AfCFEM2* gene; F1-2, verify the *AfCFEM2* deletion mutants
AfCFEM2-up-R	TCATCTTCTGTTTGACTTTGTAGTATTAAGAATTATGTAAAAA	
AfCFEM2-down-F	TTGTTAGTTGGCTTGACGGGGA	Amplify the downstream fragment (1118 bp) for *AfCFEM2* gene deletion; R1-2, verify the *AfCFEM2* deletion mutants
AfCFEM2-down-R (R1-2)	CAACTGCCGTTCGACTGTTTTTGTTTGGTGGAAGCG	
AfCFEM2-hyg-F (F2-2)	CAAAGTCAAACAGAAGATGATATTGAAGGAGCATTT	Amplify the *Hyg* resistance fragment (2121 bp) for *AfCFEM2* gene knockout, verify the *AfCFEM2* deletion mutants
AfCFEM2-hyg-R (R2-2)	CCCGTCAAGCCAACTAACAAAGAAGGATTACCTCTAAACAAGTGTACC	
AfCFEM3-up-F (F1-3)	TTCGGATCTTCCAGACATGGTAAGGTTCTTTCGCTTGG	Amplify the upstream fragment (1110 bp) of *AfCFEM3* gene; F1-3, verify the *AfCFEM3* deletion mutants
AfCFEM3-up-R	CATCTTCTGTCTCTGGAAGCTGAGTCTCAAGC	
AfCFEM3-down-F	CTTTAACCTACAACGGCACCTCCA	Amplify the downstream fragment (1104 bp) of *AfCFEM3* gene; R1-3, verify the *AfCFEM3* deletion mutants
AfCFEM3-down-R (R1-3)	CAACTGCCGTTCGACTGTTTTCGTGACATGTGATGTAAATG	
AfCFEM3-hyg-F (F2-3)	GCTTCCAGAGACAGAAGATGATATTGAAGGAGCATTT	Amplify the *Hyg* resistance fragments (2121 bp) for *AfCFEM3* gene deletion, verify the *AfCFEM3* deletion mutants
AfCFEM3-hyg-R (R2-3)	GGTGCCGTTGTAGGTTAAAGAAGGATTACCTCTAAACAAGTGTACC	
KO9810-gene-F (F3-1)	GAAGTTCTCCACTGTCATCC	Verify the *AfCFEM1* deletion mutants
KO9810-gene-R (R3-1)	GTTGCGAGCGAATCTCA	
KO2456-gene-F (F3-2)	TTGTCTCCTCCCAGCCCTCC	Verify the *AfCFEM2* deletion mutants

KO2456-gene-R (F3-2)	TTCCAGCGGCAGTCGTG	
KO9809-gene-F (F3-3)	CAGTTCTTAGTTGCGAGTGG	Verify the *AfCFEM3* deletion mutants
KO9809-gene-R (F3-3)	GGAGGTTCCGTCGATGTAG	
GPDP-F	TTCGGATCTTCCAGAGATCACCTCGGCGGGGTC	Amplify the promoter fragments (2000 bp) of *AfGpd*
GPDP-R	CGCCCTTGCTCACCATATTTAAATTTTGAATTATTGACTTTTGTCG	
GFP-F	AAATATGGTGAGCAAGGGCGAGG	Amplify the *EGFP* fragments (720 bp)
GFP-R	GCTTCACGCTGGGGAATTATTTACTTGTACAGCTCGTCCATGCC	
GPDT-F	ATAATTCCCCAGCGTGAAGCT	Amplify the terminator fragments (505 bp) of *AfGpd*
GPDT-R	TCTTCTGATGTGGAAAAGGGATTTGATCG	
hyg-F	CCCTTTTCCACATCAGAAGATGATATTGAAGGAGCATTT	Amplify the *Hyg* resistance fragments (2121 bp) for *AfCFEM* gene overexpresion
hyg-R	CAACTGCCGTTCGACAAAGAAGGATTACCTCTAAACAAGTGTACC	
OEAfCFEM1-F	AGTCAATAATTCAAAATGAAGTTCTCCACTGTCATCCTCG	Amplify the CDS fragment (393 bp) of *AfCFEM1*
OEAfCFEM1-R	GCCCTTGCTCACCATCAAGTTGCGAGCGAATCTCAA	
OEAfCFEM2-F	AGTCAATAATTCAAAATGAAGTTCTCCGCCATCGC	Amplify the CDS fragment (627 bp) of *AfCFEM2*
OEAfCFEM2-R	GCCCTTGCTCACCATCAAGAAGAAAGCAGCGACGG	
OEAfCFEM3-F	AGTCAATAATTCAAAATGAAGATCTCGACTCTATGGGCG	Amplify the CDS fragment (1845 bp) of *AfCFEM3*
OEAfCFEM3-R	GCCCTTGCTCACCATCAAAAGTAGCATTCCGAGGGTG	
pCE-Zero-ZH-F	AAGCATTTATCAGGGTTATTGTCTC	Verify the overexpression transformants
pCE-Zero-ZH-R	TGTTGTGCCCAGTCATAGCC	
AfCFEM1-5F	ATCCTCGCCGTCGCTACCT	Quantify relative transcription levels of *AfCFEM1* in *OEAfCFEM1* transformants
AfCFEM1-3R	CAGCAGCAGTGGACTTGTCCTTA	
AfCFEM2-5F	CTCTGCAAGTCCGCTGGTGT	Quantify relative transcription levels of *AfCFEM2* in *OEAfCFEM2* transformants
AfCFEM2-3R	CTCGCCGTAGGTTGGGTCA	
AfCFEM3-5F	TTTTGGCATCAGCGACTACCC	Quantify relative transcription levels of *AfCFEM3* in *OEAfCFEM3* transformants
AfCFEM3-3R	TTTTCCACTCGCAACTAAGAACTG	

Supplementary Table S2. List of primers used in RT-qPCR to determine the relative transcriptional level of *AfCFEM*1-8 genes in Δ*AfCFEM*1-3 mutants.

Gene	Primers
AfCFEM1	5F-ATCCTCGCCGTCGCTACCT
	3R-CAGCAGCAGTGGACTTGTCCTTA
AfCFEM2	5F-CTCTGCAAGTCCGCTGGTGT
	3R-CTCGCCGTAGGTTGGGTCA
AfCFEM3	5F-TTTTGGCATCAGCGACTACCC
	3R-TTTTCCACTCGCAACTAAGAACTG
AfCFEM4	5F-GCATCTGCGGCTCTAACACTTTC
	3R-CAATGGTTGGGACGATGCTACT
AfCFEM5	5F-GGCGTGGCACTGGAAACAAA
	3R-GCTAGAGCCGTCGGGCATA
AfCFEM6	5F-CACCAGCGATGTTGTTACCTTGA
	3R-AAAGCGGCACCGATGAGG
AfCFEM7	5F-CTGTTACCGTCACCGCTACCTT
	3R-CTTGTTGTTCCCGCTGGCTC
AfCFEM8	5F-CAACGATACCACCAGCAGCAC
	3R-GACAATGGCACCGAGTCCA
AfGpd	5F-TCGAGAAGCCCGCCAAGT
	3R-CAAAGGTGTCGGTCAAAGCAAT

Supplementary Table S3. Statistics of reads, phred-like quality scores, and GC content for the *A. flagrans* induced by *C. elegans* for different time points.

Sample	Raw reads	Raw bases	Clean reads	Clean bases	Error rate (%)	Q20(%)	Q30(%)	GC content (%)
AC_24_3	33286384	4896426009	33285566	4895817830	0.0251	98.04	94.15	49.77
AC_24_2	38417080	5653799583	38415950	5653095460	0.0255	97.9	93.74	49.75
AC_24_1	36946278	5453715543	36945420	5453039319	0.0256	97.84	93.66	49.81
AC_18_3	33518978	4931536115	33517774	4930818506	0.0254	97.89	93.84	49.72
AC_18_2	30182274	4470783348	30181254	4470161166	0.0255	97.86	93.74	49.46
AC_18_1	24124920	3562424962	24124174	3561899051	0.0252	97.96	94.02	49.72
AC_12_3	40419166	5959794711	40417754	5958967929	0.0253	97.95	93.97	49.58
AC_12_2	32765870	4839060659	32764934	4838437522	0.0257	97.79	93.52	49.52
AC_12_1	30189012	4478352323	30187816	4477767268	0.0254	97.93	93.8	49.61
AC_6_3	16956696	2511397890	16955858	2510946537	0.0262	97.53	93.17	49.65
AC_6_2	15378420	2284291629	15377670	2283891073	0.0256	97.75	93.64	49.56
AC_6_1	24643372	3645790482	24642290	3645200440	0.026	97.66	93.33	49.67
AC_3_3	20251382	3007385360	20250440	3006916179	0.0255	97.82	93.76	49.82
AC_3_2	21553116	3184945720	21552210	3184430450	0.0261	97.59	93.19	49.75
AC_3_1	18610510	2753430036	18609612	2752948949	0.0259	97.67	93.45	49.79
AC_0_3	15596314	2315554998	15595342	2315101816	0.0261	97.56	93.23	49.53
AC_0_2	17502882	2595310952	17501774	2594810468	0.0258	97.71	93.53	49.59
AC_0_1	20566442	3042827633	20565442	3042312508	0.0256	97.78	93.7	49.51

Supplementary Table S4. The top 20 up-regulated genes in *A. flagrans* at 18 h of interaction with *C. elegans*

Gene ID	log_2FC	*P*-adjust	Regulate	Pfam
EVM04G003750	15.54	6.28E-37	up	Egh16-like virulence factor
EVM01G006940	13.62	4.83E-28	up	Egh16-like virulence factor
EVM05G007040	13.48	2.55E-54	up	Cysteine-rich secretory protein family
EVM03G004040	13.01	2.36E-50	up	Egh16-like virulence factor
EVM03G000790	12.86	1.18E-136	up	-
EVM05G013630	12.83	5.41E-25	up	-
EVM04G003700	12.61	5.76E-143	up	Egh16-like virulence factor
EVM04G001020	12.60	2.53E-24	up	-
EVM01G010460	12.56	4.99E-24	up	Domain of unknown function
EVM02G016680	12.43	1.24E-23	up	-
EVM00G017460	12.14	1.44E-22	up	-
EVM02G016960	12.02	2.05E-21	up	Plastocyanin-like domain
EVM00G007040	11.63	3.18E-174	up	WSC domain
EVM00G018180	11.56	2.52E-20	up	CFEM domain
EVM03G010770	11.28	9.25E-26	up	Fungal cellulose binding domain
EVM02G022850	10.99	1.47E-18	up	Domain of unknown function
EVM02G002190	10.76	2.90E-17	up	-
EVM02G017030	10.68	2.03E-17	up	-
EVM00G003410	10.66	2.08E-17	up	-
EVM02G019740	10.62	5.94E-17	up	-

Supplementary Table S5. Differential expression data of 14 CFEM protein-related genes in *A. flagrans* after 18 h of interaction with *C. elegans* compared with 0 h.

Gene_id	AC_18_tpm	AC_0_tpm	log_2FC	P-adjust	regulate
EVM00G018180	707.44	0.00	11.56	2.52E-20	up
EVM01G010330	1988.52	6.18	8.30	2.24E-171	up
EVM00G018170	91.62	0.81	6.83	5.02E-64	up
EVM00G018950	298.44	36.46	3.03	1.91E-37	up
EVM03G009890	198.57	39.68	2.20	0.059710477	up
EVM00G001060	810.01	185.06	2.13	3.21E-21	up
EVM05G013090	1.00	0.49	0.98	0.659344634	up
EVM03G012640	388.31	214.10	0.85	0.000381434	up
EVM04G002530	28.43	30.43	-0.12	0.786323493	down
EVM03G000720	13.58	14.73	-0.17	0.759792386	down
EVM05G004980	0.48	0.74	-0.49	1	down
EVM02G008990	51.57	108.03	-1.10	7.48E-07	down
EVM02G012460	23.43	38.43	-0.75	0.008555989	down
EVM00G017480	8.76	23.81	-1.46	0.000996946	down

Supplementary Table S6. Differentially expressed virulence and adhesion proteins of knockout strains Δ*AfCFEM1* and Δ*AfCFEM2*.

Accession	Domain	Fold Change/P-value		Strain
		12 h	24 h	
EVM00G001030	Subtilase family	2.868/ 0.0001177	1.405/0.04061	Δ*AfCFEM1*
EVM03G007260	Subtilase family		2.236/0.001748	
EVM01G004180	Malate synthase	1.774/0.02663		
EVM01G009790	Lectin		32/0.008604	
EVM02G001720	Lectin	1.648/0.001364	1.467/0.001364	
EVM01G001350	Eukaryotic aspartyl protease	2.359/0.02352	2.436/0.0149	
EVM02G016290	Eukaryotic aspartyl protease		1.278/0.03796	
EVM00G018180	CFEM	1.00E-05/0.01949		Δ*AfCFEM2*
EVM02G005160	Egh16-like virulence factor	0.456/0.002615		
EVM00G010160	Lipase		1.00E-05/0.001851	

Supplementary Table S7. Pathways annotation of the virulence and adhesion-associated differentially expressed proteins (DEPs) in Δ*AfCFEM1* and Δ*AfCFEM2* mutants compared to WT.

Accession	Pathway ID	Strain
EVM05G005570	Autophagy - other; Autophagy - yeast	
EVM01G003940	Autophagy - yeast	
EVM02G004260	Autophagy - other; Autophagy - yeast	
EVM01G014460	Autophagy - yeast	
EVM02G004690	Autophagy - yeast	
EVM02G014760	Autophagy - yeast	
EVM01G004890	Autophagy - yeast	
EVM02G019370	Autophagy - yeast	
EVM01G013760	Autophagy - other; Autophagy - yeast	
EVM04G001370	Autophagy - yeast; Mitophagy - yeast; MAPK signaling pathway - yeast	
EVM05G008210	Mitophagy-yeast; MAPK signaling pathway - yeast	
EVM05G001700	MAPK signaling pathway - yeast	
EVM04G008110	MAPK signaling pathway - yeast	
EVM05G005210	MAPK signaling pathway - yeast	
EVM05G012510	MAPK signaling pathway - yeast	
EVM03G010580	MAPK signaling pathway - yeast	
EVM05G003510	MAPK signaling pathway - yeast	
EVM00G001630	MAPK signaling pathway - yeast	Δ*AfCFEM1*
EVM01G013310	Lysine biosynthesis	
EVM03G001900	Lysine biosynthesis	
EVM02G004940	Nitrogen metabolism	
EVM02G011400	Nitrogen metabolism	
EVM03G002490	Glycolysis / Gluconeogenesis	
EVM04G000270	Glycolysis / Gluconeogenesis	
EVM02G007540	Glycolysis / Gluconeogenesis	
EVM03G003860	Glycolysis / Gluconeogenesis; Starch and sucrose metabolism	
EVM01G007960	Starch and sucrose metabolism	
EVM00G013270	Starch and sucrose metabolism	
EVM04G005630	Starch and sucrose metabolism	
EVM03G013520	Starch and sucrose metabolism	
EVM01G007630	Starch and sucrose metabolism	
EVM00G010270	Citrate cycl	
EVM01G004400	Citrate cycl	
EVM00G016030	Citrate cycl	
EVM01G009180	Citrate cycl	
EVM00G006570	Citrate cycl	
EVM03G014130	Autophagy - other; Autophagy - yeast	Δ*AfCFEM2*
EVM04G010120	Mitophagy - yeast; MAPK signaling pathway - yeast	
